# Meeting the Needs of Older Adults with Mental Ill-Health in Non-Psychiatric Care Settings: Self-Rated Confidence in Helping and its Co-Variates within a Multiprofessional Study Sample

**DOI:** 10.1177/23337214231179819

**Published:** 2023-06-26

**Authors:** Magdalena Häger, Erika Boman, Anna K. Forsman

**Affiliations:** 1 Åland University of Applied Sciences, Mariehamn, Finland; 2 Åbo Akademi University, Vaasa, Finland; 3 Umeå University, Sweden

**Keywords:** mental health, quantitative methodology, health services research, aging

## Abstract

In this paper we sought to explore health and social care professionals’ self-rated confidence in helping older adults with mental ill-health in non-psychiatric care settings. A cross-sectional survey study was performed exploring the participants’ (*n* = 480) confidence in helping. Confidence in helping was analyzed together with background characteristics and selected explanatory variables, such as the workplace and work experience of the participants, their personal experiences of and attitudes to mental ill-health, as well as their knowledge in mental ill-health among older adults, by means of descriptive statistics and logistic regression analysis. We found that approximately half (55%) of the participants were confident in helping older adults with mental ill-health. The odds ratios for being confident in helping were significantly associated to the workplace of the professionals, professionals’ attitude to and experience of mental ill-health, and knowledge of mental health among older adults. To increase confidence in helping older adults with mental ill-health, we recommend confidence-building interventions, for example, educational programs, through which knowledge of mental health among older adults is increased and negative attitudes are challenged, especially within the context of specialist somatic healthcare.

## What this paper adds

- In exploring self-rated confidence in helping older adults with mental ill-health among health and social care professionals in non-psychiatric care settings, it seems to be knowledge in mental ill-health among older adults in particular, and not education per se, that contributes to confidence in helping
- experience of mental ill-health within family or among friends, as well as at work— especially working with older patients on a regular basis - were found to increase the likelihood of being confident in helping
- having a standpoint of equating mental and somatic health had increased likelihood of being confident in helping.

## Applications of study findings

- Possible interventions through which knowledge of and positive attitudes toward older adults with mental ill-health could be enhanced are needed
- organizational and inter-professional support might improve confidence in helping. Managers should systematically work with competence-building initiatives. Mentoring or guidance, where those with more experience share their experience are recommended initiatives
- increased collaboration with psychiatric health care services could also provide more support and thereby perhaps improve confidence in helping. Confidence is the basis for developing competence.

## Introduction

The percentage of people aged 65 years and older in the Nordic countries is expected to increase from 20% to 26% between 2020 and 2060 (Nordic Welfare Centre, 2020). Worldwide, older adults comprise the majority of health and social care service users and often have complex combinations of physical and mental ill-health needs (Andreas et al., 2017; Kujawska-Danecka et al., 2016; Malkin et al., 2019; Petrova & Khvostikova, 2021; Skoog, 2011). Older adults with mental ill-health often are referred to somatic, geriatric or primary health care, where health care professionals seldom have specialist competence in mental ill-health (Asante et al., 2022; Nordic Welfare Centre, 2020). Older adults’ undiagnosed mental ill-health can result in low life satisfaction, suffering and disability; if specialist competence within the care of older adults is lacking, quality of care and patient safety may be at risk (Skoog, 2011).

In a European study, the prevalence of mental ill-health among older adults was seen to vary between 7 to 40%, relevant to varying diagnosis criteria (Kujawska-Danecka et al., 2016). Common threats to mental health are symptoms of depression (Andreas et al., 2017; Copeland et al., 2004; Luijendijk et al., 2008; Skoog, 2011), anxiety disorders (Kujawska-Danecka et al., 2016) and mental problems related to dementia (Onyike, 2016; Wittchen et al., 2011). Mental ill-health among older adults may be challenging for health and social care professionals to recognize as the way the disorders are expressed can differ between younger and older age groups (Malkin et al., 2019; Skoog, 2011). For instance, older adults with depression are less likely to display affective symptoms and are more likely to display cognitive and somatic symptoms (Malkin et al., 2019), which can overlap with and be misinterpreted as symptoms of cognitive and/or physical disorders (Skoog, 2011). Moreover, general beliefs that symptoms of mental ill-health are a consequence of the normal aging processes may act as a barrier to older adults’ tendency to recognize and seek help, as well as hinder health and social care professionals’ identification, assessment and treatment of mental disorders (Skoog, 2011; Wuthrich & Frei, 2015). The often unrecognized and untreated mental ill-health (Melo et al., 2016; Sanders et al., 2008; Svensson & Hansson, 2017), might be explained by a gap in knowledge, which can lead to health and social care professionals’ decreased confidence in helping older adults with mental ill-health (Chuang & Kuo, 2018).

In the context of health and social care, confidence in helping can be defined as a professional’s ability to successfully accomplish a unique task in a specific context (Perry, 2011; Rosander & Jonson, 2017). Surrogate terms and concepts can be difficult to separate from one other (Perry, 2011), for example, self-confidence, self-efficacy (Holland et al., 2012), readiness (Jamieson et al., 2019), efficiency (Mcleod, 2017), preparedness (Hunter et al., 2015; Jamieson et al., 2019) and professional confidence (Holland et al., 2012; Ogawa & Nakatani, 2020). According to Perry (2011), confidence is highly individualized, complex, and rooted in a person’s self-esteem, sense of efficacy, role and contextual experiences. In health and social care, evolvement of confidence in helping is endorsed as it is essential for developing professional competence (Holland et al., 2012). Developing competence in helping is a dynamic maturing process supported through collegial cooperation, reflection in practice and receiving feedback (Holland et al., 2012). Elements that can affect confidence positively include resilience, cognitive abilities, emotional intelligence and personal characteristics (Perry, 2011) and a desire to help others is an important motivational factor with regard to confidence in helping those with mental ill-health (Crawford & Burns, 2020).

Health and social care professionals may experience a sense of inadequacy if they do not have the confidence to help older adults with mental ill-health (Melo et al., 2016; Perry, 2011; Svensson & Hansson, 2017), especially if organizational strategies for helping older adults with mental ill-health are lacking (Melo et al., 2016; Perry, 2011; Svensson & Hansson, 2017). Moreover, doubt and uncertainty may negatively impact confidence (Perry, 2011). Health and social care professionals’ attitudes, that is, their way of thinking or feeling, toward older adults with mental ill-health have even been linked to confidence in helping (Jormfeldt et al., 2013; Perry, 2011). Stigmatization of older adults with mental ill-health may have a negative effect on professionals’ helping behavior (Melo et al., 2016; Perry, 2011; Svensson & Hansson, 2017; Thornicroft et al., 2007; Williams & Tufford, 2012).

Previous work experience (Karlstedt et al., 2015; Meretoja et al., 2015), personal experience and/or a family history of mental ill-health (Al-Awadhi et al., 2017; Arvaniti et al., 2008) can increase health and social care professionals’ awareness of mental ill-health and help develop their confidence in helping. Having the professional awareness and ability to identify mental ill-health (Jormfeldt et al., 2013; Svensson & Hansson, 2017) among older adults requires knowledge of the aging process and associated mental and physical challenges (Estabrook et al., 2015; Sanders et al., 2008). Health and social care professionals’ confidence in helping, seen as an awareness and detection of mental ill-health among older adults, can even be improved through, for example, specific further education and training programs (Svensson & Hansson, 2017). For example, the Mental Health First Aid (MHFA) training program for supporting the older adult, developed in Sweden, has been shown to improve health and social care professionals’ confidence in helping even 2 years after completion of the program (Svensson & Hansson, 2017). Those health and social care professionals who have the confidence to help older adults with mental ill-health have been found to provide more high-quality care (Björkman et al., 2008; Melo et al., 2016; Svensson & Hansson, 2017).

To meet the needs and prerequisites related to demographic changes, evolving health and social care sectors, and economic constraints, the development of confidence in helping within the context of care for older adults should be improved (Nordic Welfare Centre, 2020). In this present study, we sought to explore health and social care professionals’ self-rated confidence in helping older adults with mental ill-health in non-psychiatric care settings.

Research questions: How is self-rated confidence in helping distributed among health and social care professionals in non-psychiatric care settings? Which factors (i.e., individual characteristics, workplace related factors, as well as factors related to the professionals’ knowledge and attitudes) may explain health and social care professionals’ self-rated confidence in helping in non-psychiatric care settings?

## Methods

### Design and Setting

A cross-sectional survey study, the study was conducted in Southwestern Finland in a natural geographically isolated island region with approximately 30 300 inhabitants. A total survey was conducted. All health and social care professionals in the region were invited to participate in the study. The data were analyzed using descriptive and analytical statistics including logistic regression modeling.

### Participants

**Figure 1.**
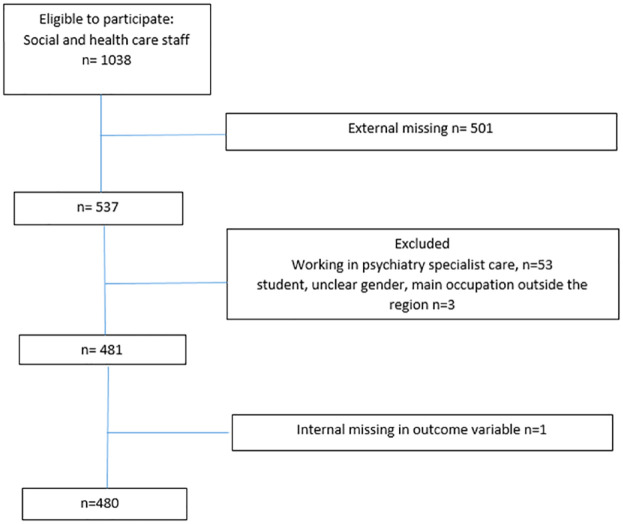
Flow-Chart of the Study Participants.

Inclusion criteria were being a currently appointed health and social care professional and caring for older adults on a regular basis. To recruit participants, a request was sent out to all operations managers within health and social care services in the region. Three operations managers declined participation, resulting in the exclusion of professionals from public dental care, prehospital emergency care and one small municipality (serving approximately 450 inhabitants). This yielded 1,038 potential participants; of these, 537 (51.7%) completed the survey. Health and social care professionals in psychiatric specialist care (*n* = 53) were excluded because the focus of the study was non-psychiatric care settings, that is, somatic health and social care. Three participants who did not meet the inclusion criteria were also excluded. In total, 480 health and social care professionals were included. For a flowchart of study participants, see Figure 1.

## Data Collection and Survey

Data collection was performed from April 2018 to May 2019 by a survey. Participants were asked to complete an anonymous (non-coded) survey (online by means of Webropol or a paper version of the online questionnaire), distributed by the employer. The survey included a variety of questions to elicit participant responses related to a dependent variable, Confidence in helping, and independent variables that may explain the probability of confidence in helping. Two rounds of pilot testing were conducted prior the data collection. Small modifications to the survey were made after the first pilot test.

### Measurements

To assess the dependent variable, Confidence in helping, eight elements (items) from a questionnaire used to evaluate the MHFA training program for supporting the older adult were included in the survey (see Table 1). The MHFA training program was first developed by Kitchner & Jorm at the beginning of 21st century in Australia (Kitchener & Jorm, 2002) and then further developed and tested in the Nordic context by Svensson and Hansson (2014, 2017). The evaluation questionnaire for the MHFA training program encompasses nine items through which self-rated confidence and skills in helping can be assessed. Of these, only eight items were included in the survey used in this present study; item nine (Information about effective treatment) was removed due to its similarity to item six (Giving information to get the right kind of help). A 5-point Likert scale ranging from “not at all” (1) to “to a very great extent” (5) was used for responses to these items. The mean values for all eight items were computed. Cronbach’s alpha was even used to test the reliability of these items, yielding an overall Cronbach’s alpha of .895. Comparison of the items revealed that item 2 had a higher mean but that the overall Cronbach’s alpha was only marginally affected (.895–.892); consequently, item 2 was not deleted.

**Table 1.** The Dependent Variable, Confidence in Helping, Assessed Using Eight Items from the Evaluation Questionnaire of the MHFA Training Program for Supporting the Older Adults (Means and Standard Deviations).

Mental Health First Aid (MHFA) item	*M*	*SD*
Making contact with an older adult with mental ill-health	3.93	0.927
Taking time to listen and listen nonjudgmentally to an older adult	4.34	0.766
Being aware of how a sad and a depressed older adult communicates	3.56	0.999
Asking if the older adult has suicidal thoughts	3.26	1.278
Giving information to an older adult about how to get the right kind of help	3.27	1.124
Recognizing signs of mental ill-health in an older adult	3.45	0.882
Assessing seriousness of mental illness in an older adult	3.21	1.011

The independent variables included participant background characteristics (gender, marital status, educational level) and explanatory variables, for example, workplace, work experience, personal experiences of mental ill-health, attitudes to mental ill-health, and knowledge of mental ill-health among older adults (see Table 2). To explore participants’ personal experiences of mental ill-health, three questions on mental ill-health history inspired by Kitchener and Jorm (2002) were included in the survey. To explore participants’ attitudes to mental ill-health, one statement from the Community Attitudes toward Mental Illness (CAMI) scale was included in the survey: “Mental illness is an illness like any other.” A 6-point Likert scale ranging from “totally disagree” (1) to “totally agree” (6) was used for this item. To explore participants’ knowledge of mental ill-health among older adults, various statements from a 19-item scale developed by Ahlin Åkerman 2013 (Svensson & Hansson, 2017) were included in the survey. For example, “Depression is the most common type of mental ill-health problem at an advanced age,” “It is common that older adults have a mix of both depression and anxiety,” and “It is assumed that older adults are more vulnerable to post traumatic stress compared to younger persons.” The response alternatives for these items were “correct” (1 point), “not correct” (0 point) or “do not know” (0 point) and a score for these was tallied to form a “Knowledge score” ranging from 0 to 19, with a higher score indicating better knowledge.

**Table 2.** The Distribution of the Independent Variables by Confidence in Helping, Expressed as “Confident,” “Not Confident,” (N, %).

Variable (*N* total)*	Confident *N* = 265 (55.2)	Not confident *N* = 215 (44.8)	Total	*p*-value
	*N* (%)	*N* (%)	*N* (%)	
Gender (N = 480)				*p* = .476^a^
Women	242 (54.6)	201 (45.4)	443 (92.3)	
Men	23 (62.2)	14 (37.8)	37 (7.7)	
Marital status (N= 476)				*p* = .762^a^
Living alone^c^	62 (57.4)	46 (42.6)	108 (22.7)	
Living in a relationship^d^	203 (55.2)	165 (44.8)	368 (77.3)	
Educational level (N= 471)				***p*** < **.001**^a^
Lower^e^	132 (62.9)	78 (37.1)	210 (44.6)	
Medium^f^	101 (46.3)	117 (53.7)	218 (46.3)	
Advanced^g^	29 (67.4)	14 (32.6)	43 (9.1)	
Workplace/setting (N= 480)				***p*** < **.001**^a^
Senior health and social care^h^	133 (68.2)	62 (31.8)	195 (40.6)	
Specialist care^i^	89 (41.0)	128 (59.0)	217 (45.2)	
Primary care^j^	43 (63.2)	25 (36.8)	68 (14.2)	
Work experience (N= 480)				*p* = .171^a^
0–2 years	12 (46.2)	14 (53.8)	26 (5.4)	
3–10 years	62 (49.6)	63 (50.4)	125 (26.0)	
>10 years	191 (58.1)	138 (41.9)	329 (68.5)	
Experience of mental ill-health				
No experience	220 (56.1)	172 (43.9)	392 (81.7)	*p* = .465^a^
Own	45 (51.1)	43 (48.9)	88 (18.3)	*p* = .465^a^
Within family/among friends	163 (61.0)	104 (39.0)	267 (55.6)	***p*** = **.005**^a^
Work-related	171 (58.4)	122 (41.6)	293 (61.0)	*p* = .100^a^
Attitude to mental ill-health: Mental ill-health is comparable to somatic illness (N= 479)				*p* = .307^a^
Disagree	26 (45.6)	31 (54.4)	57 (11.9)	
Agree	48 (56.5)	37 (43.5)	85 (17.7)	
Totally agree	190 (56.4)	147 (43.6)	337 (70.4)	
	M (SD)	M (SD)	M (SD)	
Knowledge score (461)	10.5 (2.7)	9.7 (2.7)	10.13 (2.72)	***p*** = **.003**^b^

* *N* total may vary due to internal missing.

a Chi-Square Test (statistically significant values marked in bold).

b *t*-Test.

c Living alone (divorced/unmarried/widowed/other).

d Living in a relationship (married/civil partnership/civil partnership, separate abode).

e Lower (basic education, high school, practical nurse).

f Medium (vocational degree).

g Advanced (Master’s degree, specialist nurses, physicians).

h Senior health and social care and support (home care, facility, adult day services, assisted-living, care for older persons/persons with disabilities, municipal social services).

i Specialist care (hospital wards, emergency unit, outpatient unit, intensive care unit, operations).

j Primary care (health care centers, home care).

### Analysis

The data were analyzed using SPSS version 27.0 (IBM Corp, 2020). Using the data derived from analysis of the dependent variable, Confidence in helping, quartiles were used to place the participants into groups, seen as “confident” (score of 3, 4 or 5 on all eight items) or “not confident.” The Chi-square test and Independent samples t-test were then used to compare the independent variables between the “confident” and “not confident” groups. The first analysis revealed the emergence of three subgroups. Hence Fisher’s exact test was used to perform three pairwise subgroup analyses, alongside Bonferroni correction. In order to fit the formulated aim and research questions, and due to data characteristics (i.e., ordinal scale level of measurements for the dependent variable, data skewness), binary logistic regression analysis was conducted to assess groupwise differences between participants rating themselves as confident or not confident in helping older adults with mental health problems controlling for the independent variables and background variables alike. Predicted probabilities of confidence in helping were determined by odds ratio (OR) and confidence intervals (CI). Prior to the regression analysis, we checked for multicollinearity. A correlation was found between the variables Profession and Educational level; thus the variable Profession was excluded prior to the regression analysis. The Nagelkerke *R*^2^ was examined to assess the percent of variance accounted for by the independent variables. A p-value of <.05 was regarded as statistically significant.

## Results

The vast majority of the 480 included health and social care professionals were women (92.3%) with a lower or medium educational level (44.6% and 46.3%, respectively) whose workplace was specialist care (45.2%) or senior health and social care (40.6%). Most had more than 10 years of work experience (68.5%), work-related experience of mental ill-health (61.0%), and experience of mental ill-health within family/among friends (55.6%).

In total, 55.2% of the participants were in the “confident” group (*n* = 265). Participants’ self-rated confidence was highest for the items “Taking time to listen and listen nonjudgmentally” (*M* = 4.34) and “Making contact with a person with mental ill-health” (*M* = 3.93). Participants’ self-rated confidence was lowest for the items “Assessing the seriousness of mental illness” (*M* = 3.21) and “Asking if the person has suicidal thoughts” (*M* = 3.26). See Table 1.

With respect to the variables Educational level, Workplace, Experience of mental ill-health and the mean Knowledge score significant differences were seen between the “confident” group and the “not confident” group. For further information, see Table 2.

Logistic regression was performed to study the impact of the independent variables on the likelihood of being in the “confident” or the “not confident” group. As seen in Table 3, the variable Workplace was significantly associated with “confident.” Those whose workplace was specialist care had lower OR for “confident” (0.26 95% CI [0.14, 0.48]), when compared to those whose workplace was senior health and social care (the reference group). For the variable Experience of mental ill-health, the OR was significantly higher for those with experience within family/among friends (1.83 95% CI [1.20, 2.80]) and work-related experience (1.75 95% CI [1.12, 2.71]). There was no significant association between likelihood of being in the “confident” group and the socio-economic background variables Gender, Marital status, Work experience and/or Education level.

**Table 3.** Odds Ratios (ORs) and Their 95% Confidence Intervals (CIs) of Self-Rated Confidence in Helping among Health and Social Care Professionals (*N* = 480).

Variable	OR, 95% CI
Gender	
Women	1
Men	1.46 [0.65, 3.27]
Marital status	
Living alone	1
Living in a relationship	0.84 [0.51, 1.39]
Educational level	
Lower	1
Medium	0.85 [0.46, 1.59]
Advanced	1.83 [0.74, 4.56]
Workplace/setting	
Senior health and social care	1
Specialist care	**0.26** [**0.14**, **0.48**]
Primary care	0.61 [0.27, 1.40]
Work experience	
0–2 years	1
3–10 years	0.73 [0.28, 1.89]
>10 years	1.53 [0.62, 3.79]
Experience of mental ill-health	
No experience	1
Own	0.69 [0.40, 1.19]
Within family/among friends	**1.83** [**1.20**, **2.80**]
Work-related experience	**1.75** [**1.12**, **2.71**]
Attitude to mental ill-health: Mental illness is comparable to somatic illness	
Disagree	1
Agree	**2.41** [**1.11**, **5.25**]
Totally agree	**2.36** [**1.21**, **4.60**]
Knowledge score	**1.09** [**1.01**, **1.18**]

Statistically Significant Values Marked in Bold.

For the variable Attitude to mental ill-health: Mental illness is comparable to somatic illness, the OR for “confident” was significantly higher for an “agree” (2.41 95% CI [1.11, 5.25]) or “totally agree” (2.36 95%, CI [1.21, 4.60]) response compared to a “disagree” response (the reference group). Regarding knowledge of mental ill-health among older adults, expressed as the variable Knowledge score, the OR for “confident” (1.09 95% CI [1.01, 1.18]), was seen to be associated with each point increase in overall score. See Table 3.

## Discussion

In this study, we sought to explore health and social care professionals’ self-rated confidence in helping older adults with mental ill-health in non-psychiatric care settings. From the findings, we discerned that health and social care professionals in specialist care had lower odds of confidence in helping older adults with mental ill-health when compared to professionals in senior health and social care. This might be linked to workplace setting characteristics. Professionals in senior health care encounter and gain experience of the targeted patient group on a regular basis (Seitz et al., 2010) and routinely working with older adults with mental ill-health may contribute to confidence in helping (Bing-Jonsson et al., 2015; Cremonini et al., 2018). In addition, professionals in senior health and social care have been found to possibly have a more positive attitude toward mental ill-health than professionals in somatic specialist care (Cremonini et al., 2018). One can question whether a more holistic perspective, in which both *soma* and *psyche* are embodied, is more commonly implemented in senior health and social care than in somatic care. Given that a holistic perspective should be included in all health care (Holmberg et al., 2020; Zetterberg et al., 2022), such a discrepancy can be considered problematic.

Further, workplace setting characteristics include organizational factors (McCabe et al., 2017) and workplace attributes (Perry, 2011), which have been linked to professionals’ confidence or lack thereof. Researchers in previous studies have found that professional confidence can be increased through reflection, feedback (Holland et al., 2012), learning together (Bing-Jonsson et al., 2015), interprofessional collaboration and support (McCabe et al., 2017) or collective support (Rosander & Jonson, 2017). This is consistent with the ideal organization’s vision of the learning organization, in which people continually expand their capacity, visionary thinking patterns are nurtured, the collective aspiration is set free, and there is an ongoing process in learning how to learn together (Senge, 1990). Unfortunately, the hierarchy that limits feedback between colleagues is a common characteristic of many organizations, including the public health system (McGowan et al., 2013). A learning organization, however, manage the social dynamics within their team (Doyle et al., 2021). Moving toward learning organization ideals may not only increase professional’s confidence in helping, but is also associated with organizational commitment and job satisfaction (Jeong et al., 2007).

We also saw that those health and social care professionals who expressed that mental ill-health was comparable to other forms of (physical) ill-health were more probable to be confident in helping older adults with mental ill-health. Organizational and cultural factors may impact health and social care professionals’ attitudes (Harangozo et al., 2014; Heim et al., 2018). A negative attitude toward those with mental ill-health (Björkman et al., 2008; Han & Richardson, 2015; Melo et al., 2016; Thornicroft et al., 2007; Williams & Tufford, 2012) may negatively impact professionals’ confidence in helping (Chuang & Kuo, 2018; Corrigan, Druss, & Perlick, 2014; Konnert et al., 2019; Wuthrich & Frei, 2015), and might even lead to the manifestation of a non-respectful and/or discriminatory manner toward those with mental ill-health during the provision of care (Corrigan, Mittal et al., 2014; Melo et al., 2016; Thornicroft et al., 2007). Some researchers have even seen a significant relationship between perceived discrimination and changes in depressive symptoms among older adults and emphasize the importance of care professionals being aware of and taking such into account in practice (Han & Richardson, 2015).

We also found that health and social care professionals’ own experiences of mental ill-health were not associated with increased confidence in helping. Other researchers have found that doctors’ own experiences of significant illness (e.g., anxiety, depression, substance abuse) appear to have little effect on subsequent care for their patients (Fox et al., 2009) but may result in qualitative changes to their communication style (Hall et al., 2018). However, confidence in helping was significantly higher for those with experience of mental ill-health within family/among friends or work-related experience. Also other researchers have seen that care professionals’ personal experience and/or a family history of mental ill-health may reduce negative attitudes toward mental ill-health (Arvaniti et al., 2008) or indicate an attitude of benevolence (Al-Awadhi et al., 2017). Personal and/or familial experiences of mental illness may possibly lead to reflection on own professional approach and the development of a holistic professional approach (Oates et al., 2018). This may be a valuable resource in caring in a learning organization.

We furthermore found that more knowledge of older adults’ mental ill-health was associated with higher odds for being confident in caring for older adults with mental ill-health. Education can increase health care professionals’ adapted knowledge and enable the provision of quality care to those with mental ill-health (Konnert et al., 2019). Knowledgeable professionals may even have a more positive attitude toward those with mental ill-health, which can lead to enhanced recovery (Cremonini et al., 2018). The combination of interventions for and contact with patients with mental ill-health may increase knowledge and facilitate a professional attitude that in turn supports confidence in treating those with mental ill-health (Henderson et al., 2014). Researchers have found both education and experience can increase care professionals’ knowledge (Lejonqvist et al., 2012) and that knowledge of mental ill-health may increase nurses’ confidence in helping older adults with mental ill-health (Chuang & Kuo, 2018).

Nevertheless, as seen in this study and in line with another study (Chuang & Kuo, 2018), neither length of work experience nor educational level were significant with regard to confidence in helping older adults with mental ill-health. While knowledge of mental ill-health is important, knowledge alone does not enhance professionals’ confidence in helping (McCabe et al., 2017). Health and social care professionals may demonstrate confidence in helping despite having less knowledge of mental ill-health which may lead to negative care outcomes (Chuang & Kuo, 2018). Experience of taking care of older adults with mental ill-health together with professional, educational interventions can increase confidence in helping older adults with mental ill-health (Chuang & Kuo, 2018; Henderson et al., 2014; Jensen et al., 2016). Educational interventions might provide an effective way to decrease stigma against those with mental ill-health, especially for those general health care professionals with little or no formal mental health training (Henderson et al., 2014). One such educational intervention is the internationally well-known MHFA training program, which has been found to be effective in increasing awareness and knowledge of mental ill-health—even among those professionals with an educational background and/or further training in mental ill-health (Svensson & Hansson, 2014). The MHFA training program has been shown to improve experienced confidence in helping as well as knowledge about what to do and how to act when encountering those with mental ill-health (Jensen et al., 2016; Svensson & Hansson, 2014).

Confidence-building interventions are needed to safeguard multi-professional competence in meeting and supporting the needs of the growing older population within various care settings. To ensure safe care, managers in health and social care should systematically work to develop competence (Bing-Jonsson et al., 2015). Mentoring or guidance, where those with more experience share their experience with those with less experience and security (Holland et al., 2012; Meretoja et al., 2015), is even recommended. Lastly, increased collaboration with psychiatric health care services could also provide health and social care professionals with more support and thereby perhaps improve their confidence in helping those with mental ill-health.

## Strengths and Limitations

A strength of this study is that we performed a regional total population study encompassing a multiprofessional and heterogeneous sample of health and social care professionals’ self-rated confidence in helping. Being a multidimensional concept, confidence in helping is challenging to measure. Nonetheless, the psychometrics of the measurement used was adequate and in line with previous studies, indicating reliability of our findings. Our use of binary logistic regression facilitated comparison between groups and the revealing of support or risk factors related to confidence in helping.

However, variable dichotomization as performed in this study may result in the loss of nuances; despite attempts to ensure adequate groupings (e.g., the use of first quartile as cut-off), some participants may have been erroneously grouped as “confident” or “not confident.” Also, some of the between-group differences found were relatively small. We are aware of the limitations related to logistic regression analysis demanding artificial grouping of the study sample, which may risk that nuances of some of the variables are lost during the recoding process. On the other hand, not dividing the (limited sized) sample into groups—or using to many groups—could have led to losing statistical power in the analysis (Sperandei, 2014). Background data for those who choose not to participate was not included, which may indicate bias. Further, the electronic survey format could have been a barrier to respond for the target group under study. Nevertheless, the results can be considered generalizable, albeit with some caution.

## Conclusions

In total, 55.2% of the multiprofessional sample were assessed to be confident in helping older adults with mental ill-health. As seen in the findings, it is knowledge and not education per se that contributes to health and social care professionals’ confidence in helping older adults with mental ill-health. Also, experience of mental ill-health within family or among friends and workplace experience, especially working with older patients on a regular basis as seen in senior health and social care, were seen to increase likelihood of being confident in helping older adults with mental ill-health. The findings further showed that having a standpoint of equating mental and somatic health increased likelihood of being confident. We recommend the implementation and evaluation of confidence-building interventions, as educational programs on mental health of the older adult and collaborative learning to increase knowledge and challenge attitudes toward mental health are recommended, especially in specialist somatic healthcare. This, to increase confidence in helping older adults with mental ill-health.

In future studies the use of a longitudinal approach is warranted. Further, interventions through which health and social care professionals’ knowledge of and positive attitudes toward older adults with mental ill-health can be increased should be explored. For example, an evaluation of the MHFA training program for supporting the older adult may yield deeper understanding of health and social care professionals’ confidence in helping and their experiences of caring for older adults with mental ill-health.
